# Phenotypic and Genotypic Eligible Methods for *Salmonella* Typhimurium Source Tracking

**DOI:** 10.3389/fmicb.2017.02587

**Published:** 2017-12-22

**Authors:** Rafaela G. Ferrari, Pedro H. N. Panzenhagen, Carlos A. Conte-Junior

**Affiliations:** ^1^Molecular and Analytical Laboratory Center, Department of Food Technology, Faculty of Veterinary, Universidade Federal Fluminense, Niterói, Brazil; ^2^Food Science Program, Chemistry Institute, Universidade Federal do Rio de Janeiro, Rio de Janeiro, Brazil; ^3^National Institute of Health Quality Control, Fundação Oswaldo Cruz, Rio de Janeiro, Brazil

**Keywords:** phage typing, antimicrobial resistance profile, PFGE, MLVA, MLST, CRISPR, Ribotyping, WGS

## Abstract

Salmonellosis is one of the most common causes of foodborne infection and a leading cause of human gastroenteritis. Throughout the last decade, *Salmonella enterica* serotype Typhimurium (ST) has shown an increase report with the simultaneous emergence of multidrug-resistant isolates, as phage type DT104. Therefore, to successfully control this microorganism, it is important to attribute salmonellosis to the exact source. Studies of *Salmonella* source attribution have been performed to determine the main food/food-production animals involved, toward which, control efforts should be correctly directed. Hence, the election of a ST subtyping method depends on the particular problem that efforts must be directed, the resources and the data available. Generally, before choosing a molecular subtyping, phenotyping approaches such as serotyping, phage typing, and antimicrobial resistance profiling are implemented as a screening of an investigation, and the results are computed using frequency-matching models (i.e., Dutch, Hald and Asymmetric Island models). Actually, due to the advancement of molecular tools as PFGE, MLVA, MLST, CRISPR, and WGS more precise results have been obtained, but even with these technologies, there are still gaps to be elucidated. To address this issue, an important question needs to be answered: what are the currently suitable subtyping methods to source attribute ST. This review presents the most frequently applied subtyping methods used to characterize ST, analyses the major available microbial subtyping attribution models and ponders the use of conventional phenotyping methods, as well as, the most applied genotypic tools in the context of their potential applicability to investigates ST source tracking.

## Introduction

Salmonellosis is considered one of the most important zoonosis and one of the major worldwide foodborne diseases (Petrovska et al., 2016). Among diarrheal disease agents, *Salmonella* spp. is the foodborne pathogen that accounted more deaths, which a 59,000 of 420,000 casualties until 2015, due to foodborne hazards (Scallan et al., 2011; WHO, 2015). Nowadays, over than 2,600 serotypes of *Salmonella enterica* have been identified, and *Salmonella* Typhimurium (ST), including monophasic variant *S*. 4,[5],12:i:-, is the second most frequent isolated serotype after Enteritidis in worldwide (Hendriksen et al., 2011). Morbidity and mortality have been commonly associated with ST and the infections are considered sporadic and can be occurred from several sources via different pathways, including environmental and direct contact with animals (EFSA and ECDPC, 2015; WHO, 2015). However, the most common source is the consumption of contaminated food, with 86–95% of cases (Majowicz et al., 2010). Several studies have reported a variety of different foods where *S*. Typhimurium was isolated, including pork (Norton et al., 2012; Alt et al., 2015; Arnedo-Pena et al., 2016), beef (Guillier et al., 2013), fruits and vegetables (Nillian et al., 2011; Pui et al., 2011), as well as, in pigs, pork, cattle, and beef, this serotype was also identified as the most occurrence (Barco et al., 2012; EFSA and ECDPC, 2016). In contrast to what occurs with *S*. Enteritidis, where eggs are the main route of access to humans (Alcocer and Oliveira, 2003; Kottwitz et al., 2013; Mughini-Gras et al., 2014a) the actual associated pathway through which ST finally affects a human host still needs to be clarified (Mughini-Gras et al., 2014a).

To prioritize and implement correct targeted controls in the food chain, it is crucial to attribute human ST infections to specific sources (Hald et al., 2012). Source attribution is a subject area of epidemiological research that is gaining importance and more viability by incorporating a growing number of methodological approaches and data types (Pires, 2013). The characterization of a bacteria beyond the species and/or subspecies level is defined as bacteria subtyping (Barco et al., 2013). By analyzing and comparing how often a given pathogen occurs in food and comparing it to those isolated subtypes of humans and animals and/or animals production, it may be possible to make inferences about the sources of human infections (Hyytiä-Trees et al., 2007). The strains isolated from the originating source are expected to have same or more similar subtypes responsible for human food infections than those isolated from unrelated sources (EFSA, 2013). To choose a subtyping method of a given pathogen, it is necessary to know the epidemiological context, in which the method will be used, as well as the time and geographical scale (EFSA, 2013). For researchers and/or epidemiologists to achieve this task, frequency-matched models based on microbial subtyping have been used widely for *Salmonella*, as: Dutch model, Hald (or Danish) model (Pires and Hald, 2009; Mughini-Gras et al., in press) or Asymmetric island models (Pires and Hald, 2009; David et al., 2013). The principle behind these methodologies is the comparison of the subtypes in putative sources with the subtypes identified in human samples (EFSA, 2013). The Dutch model does not account for differences in the ability of subtypes and sources to infect humans (Mughini-Gras and van Pelt, 2014). It always provides attributions in a rather proportional and straight way and makes the arguable assumption of an equal impact of the different subtypes and sources to the human population (Barco et al., 2013, 2015; Mughini-Gras et al., 2014b). The Danish model compares the number of human cases caused by different *Salmonella* subtypes with their prevalence in different food sources, but this model also incorporated bacterial and food source dependent factors. This model, likewise, takes into consideration the origin of human cases (domestic or travel related) and whether the cases were sporadic or from an outbreak (Barco et al., 2013). Another fundamental purpose is to explain how virulence and other phenotypic traits evolve in microorganisms over time, and thereby contribute to the survival of the organism and disease severity and spread (EFSA, 2013). So, in this context, multiple phenotype and genotype *Salmonella* methods have been developed to efficiently detect the cases of human salmonellosis and your possible harm (EFSA, 2008).

Typing techniques, as phenotypic or genotypic, should be able to type all the isolates of a study (high typeability) and discriminate, at an appropriate level, those isolates (discriminatory power) (van Belkum et al., 2007). Reproducibility and typeability are comparatively easy to quantify and are often resulted as simple percentages; however, the ability of a typing method to distinguish between unrelated strains is named discriminatory index (DI) (Hunter and Gaston, 1988). In a typing method, the index should be 1.00 to be considered ideal, but, in an experimental study, it should be at least in the order of 0.95 (Hunter and Gaston, 1988). Moreover, the calculations of confidence interval should be accompanied by the diversity index (van Belkum et al., 2007). Finally, these approaches should be able to detect markers sufficiently stables to ensure that it is still possible to identify all epidemiologically related isolates in, e.g., a long-term outbreak, where some genetic divergence would be expected (Petersen et al., 2011). A high degree of reproducibility of a method will provide reliable results of the method making it possible to be included in databases and analyzed by software computer (van Belkum et al., 2007).

The traditional typing methods include phenotype-based approaches, such as serotyping, phage typing, biochemical profiling and antimicrobial resistance profile (Olsen et al., 1993; Herikstad et al., 2002). Organisms grouped according to their similarity due to the result of the expression of their genotypes is a practical definition of phenotyping approaches (van Belkum et al., 2007). The antigenic characterization of the organism by identifying the flagellar (H) and somatic (O) antigens through specific antisera reactions is called serotyping (Olsen et al., 1993; Herikstad et al., 2002; Eriksson et al., 2005). Numerous researchers have recently proposed a different molecular typing to replace conventional *Salmonella* serotyping isolates (Ranieri et al., 2013), e.g., repetitive sequence-based PCR (Wise et al., 2009), microarrays (Fang et al., 2010), pulsed field gel electrophoresis (Kérouanton et al., 2007; Zou et al., 2010) and, multilocus sequence typing (Achtman et al., 2012). However, all these techniques need further validation before can be adopted internationally as serologically type (EFSA, 2008). In the clinical microbiology field, the antibiogram typing has been considered for many years the election method to identify possible cases of bacterial cross-transmission in healthcare institutions (Tenover et al., 1997). Beyond the serotype level, both antimicrobial susceptibility profiling and phage typing are the traditional methods for *Salmonella* phenotyping (Jeoffreys et al., 2001). Their discriminatory power is sufficient to source attribute ST and are implemented as a screening of an investigation by computing results using frequency-matching models, as above described. Despite useful, these subtyping methods still often require being complemented by molecular DNA analysis, such as ribotyping and pulsed field electrophoresis (PFGE) (Wattiau et al., 2011).

Genotyping methods, such as the restriction enzyme digestion-based methods (i.e., PFGE) and DNA sequence based methods, i.e., multi-locus variable-number tandem repeat analysis (MLVA); multi-locus sequence typing (MLST); ribotyping; clustered regularly interspaced short palindromic repeats (CRISPR) (Mojica et al., 2000); and, whole genome sequencing (WGS), are technologies that study the bacterial DNA instead of their phenotypic characteristics (Foley et al., 2006). All these technics, due to its specificity, enable outbreaks to be detected and controlled at an earlier stage, as well as, enhance the detection of geographically dispersed outbreaks (Mughini-Gras et al., 2014b). However, each method has their own advantages and limitations concerning speed, cost, strengths, and sensitivity (Zou et al., 2013). Indeed, such techniques may fill the lackness left by phenotypic approaches and provide a better accuracy, crucial for epidemiological investigations (Hopkins et al., 2011; Liu et al., 2016). Currently, due to the stability of the generated profiles, the discriminatory power and reproducibility of the results, PFGE is considered the golden standard for genotyping of *Salmonella* and it is the only universal molecular method appropriate for all *Salmonella* serotypes (Wattiau et al., 2011; Mughini-Gras et al., 2014b). In contrast, MLVA is serotype specific and was developed for ST (Lindstedt et al., 2003). This method has a higher discrimination power and is widely used for surveillance and outbreak studies of ST in comparison with PFGE (EFSA, 2013).

Several genotypic methods have been used to determine how closely related ST are and provide enough data to track their sources. However, this review is strictly aimed to examine the molecular typing techniques that are very recently eligible by the scientific community and how they may be applied to study ST when implicated as foodborne pathogens. We focused on those methods that have a substantial impact on public health and there is a growing interest concerning to epidemiological use for tracing *Salmonella* Typhimurium. Thus, a thorough understanding of the advantages and limitations of such typing techniques is crucial in the choice of the appropriate approach to best define a pathogen responsible for an outbreak.

## Phenotypic methods

Immunological reactions (serotyping), biochemical markers (biotyping), or bacteriophage susceptibility (phage typing) are the phenotypic technics commonly used to subtype a bacterium. Although nowadays genotyping has become routine, phenotyping technics, as serotyping and phage typing, sometimes combined with antimicrobial resistance profiling, are the methods of choice for classical *Salmonella* source attribution (EFSA, 2008). In particular cases, these approaches possess enough discriminatory power to exploit the relative source of the different strains (EFSA, 2008). Naturally, tracing studies depends on typing technics that should have sufficient discriminatory power to recognize links between putative sources and human isolates. Besides, it is necessary that such techniques have adequate discriminatory indices once the real epidemiological association between strains might be lost (Barco et al., 2015).

### Phage typing

*Salmonella* Typhimurium phage typing scheme was first described in 1943 and defined 12 types with 11 phages (Felix, 1956). It was completed in 1959 to 34 types using 29 phages (Callow, 1959) and more recently an extended phage typing system distinguished 207 definitive types using 34 phages (Anderson et al., 1977). Nodaway, phage typing allowed to discriminated over 300 phage types and, together with antimicrobial susceptibility analyses, detected international outbreaks including some ST multi-drug resistant (MDR) clones dissemination (Threlfall, 2000; Liebana et al., 2002b; Perron et al., 2007).

Since the middle of the 90s, for source attribution and outbreak investigations, phage typing has proven to be a valuable tool for strain characterization and the results obtained have been used in surveillance (Baggesen et al., 2010). Phage typing is mainly characterized by the ability of a given phage to lyse a particular strain (Felix, 1956). Capacity to infect and lyse a bacteria cell depends on the phage molecular characteristics and their receptor presents on the surface of the bacteria (Schmieger, 1999). The significant advantage of this technique is conferred by the noncomplex implementation, which requires only basic laboratory equipment. However, due to the low number of available phages, this method is limited, as well as, it needs standardization to guarantee comparability among laboratories and assurance the reliableness. Additionally, phage typing requires experience in interpretation of results, a well-maintained phage library and accurate methodology (Threlfall and Frost, 1990). Indeed, different results among the laboratories have confused past outbreaks investigations (Ross and Heuzenroeder, 2005). Thus, to keep a major role in the control of the ST outbreaks and surveillance, this technique needs the effort to make the system available to more internationally laboratories (Baggesen et al., 2010). Despite the limitations, when phage typing is combined with antimicrobial resistance in initial studies of the potential relatedness among strains, it stills represents a valuable tool, especially to differentiates related strains and identification of emergence of MDR ST phage types (Barco et al., 2012).

Phage typing approach, in some particular cases, could be useful as tool to indicate whether isolates belong to biphasic ST instead of *S*. 4,[5],12:i:-. Studies showed a higher diversity among ST isolates compared to *S*. 4,[5],12:i:- (Dionisi et al., 2009; Soyer et al., 2009; Barco et al., 2012, 2015) and, such finding was attributed mostly to some phenotypic characters as phage type. Many phage types were found specifically in ST isolates and a restricted number was more indicative of *S*. 4,[5],12:i:- (Barco et al., 2012). However, it is important to take into account that changes in the dominating clones by a spatial or temporal evolution may occur and could generate different results due to phage conversion (Cho et al., 2007; Barco et al., 2012). In this context, different mechanisms have been identified as potential causes of phage conversion in *Salmonella* isolates such as expression of temperate phages, acquisition or loss of plasmids and mutation of genes encoding lipopolysaccharide (Olsen et al., 1993). Thus, phage typing cannot be used as the definitive method to differentiate *S*. 4,[5],12:i:- from ST serotypes (Barco et al., 2012). Fabre et al. (2012), for example, compared the DI of different approaches to differentiate between *S*. 4,[5],12:i:- and ST (Table 1). However, they only performed phage typing technic to ST isolates, which gave 14 different profiles with a DI = 0.74, in this study the DI of this phenotypic approach was lower than molecular technics to ST isolates.

**Table 1:** Different subtyping methods of *Salmonella* Typhimurium strains and respectively discriminatory index.

**Source**	**Number of strains**		**Serotype**	**Phenotype methods**	**DI (95% CI)**	**Genotype methods**	**DI (95% CI)**	**References**
Swine	32		Typhimurium DT104	PP	0.76	*Spe*I-PFGE *Xba*l-PFGE *Bln*I-PFGE 3enz.PFGE PP-*Spe*I-PFGE PP-*Xba*I-PFGE PP-*Bln*I-PFGE PP-3enz.PFGE RAPD (23L/OPB15/B) RAPD (12 primers)	0.520 0.332 0.599 0.796 0.853 0.794 0.893 0.909 0.673 0.778	Malorny et al., 2001
Swine	40		Typhimurium	PT AMR	0.628 0.579	AFLP *Xba*l-PFGE Rep PCR	0.939 0.925 0.421	Gebreyes et al., 2006
Swine Asymptomatic (85) Swine Disease-associated (44)	129		Typhimurium DT 104	AMR	0.77 0.80	*Xba*l-PFGE *Spe*I-PFGE *Xba*l-*Spe*I PFGE *Xba*l-PFGE *Spe*I-PFGE *Xba*l-*Spe*I PFGE	0.87 0.89 0.96 0.72 0.85 0.93	Perron et al., 2007
Animals	78	41 37	Typhimurium Non-DT 126 Typhimurium DT 126	–	–	10-loci MLVA MAPLT 10-loci MLVA MAPLT	0.913 0.880 0.829 0.405	Ross et al., 2009
Egg	54		Typhimurium	–	–	*Xba*l-PFGE *Spe*I-PFGE	0.60 0.71	Rivoal et al., 2009
Human	183 203		Typhimurium Typhimurium (closely related)	–	–	*Xba*l-PFGE 4-loci MLVA 5-loci MLVA 8-loci MLVA 16-loci MLVA *Xba*l-PFGE 4-loci MLVA 5-loci MLVA 8-loci MLVA 16-loci MLVA	0.995 (0.992–0.998) 0.997 (0.995–0.999) 0.997 (0.996–0.999) 0.997 (0.996–0.999) 0.997 (0.996–0.999) 0.846 (0.825–0.866) 0.976 (0.966–0.985) 0.977 (0.968–0.986) 0.980 (0.972–0.988) 0.981 (0.973–0.989)	Chiou et al., 2010
Human Bovine Human (120) Bovine (62)	28 182		4,5,12:i:_ Typhimurium	–	–	4-loci MLVA *Xba*l-PFGE MLVA- PFGE PFGE-AMR 4-loci MLVA *Xba*l-PFGE MLVA- PFGE PFGE-AMR 4-loci MLVA *Xba*l-PFGE MLVA- PFGE PFGE-AMR	0.910 (0.843–0.977) 0.833 (0.711–0.955) 0.971 (0.940–1.000) 0.844 (0.717–0.971) 0.994 (0.990–0.999) 0.975 (0.960–0.990) 0.997 (0.994–1.000) 0.990 (0.979–0.997) 0.976 (0.959–0.993) 0.938 (0.910–0.966) 0.992 (0.984–0.999) 0.980 (0.967–0.994)	Hoelzer et al., 2010
Human outbreaks	100		DT 101	–	–	STTR9 STTR5 STTR6 STTR10 STTR3	0.131 (0.044–0.219) 0.797 (0.757–0.836) 0.059 (0.000–0.122) 0.078 (0.005–0.150) 0.131 (0.044–0.219)	Dyet et al., 2011
	37		DT 104			STTR9 STTR5 STTR6 STTR10 STTR3	0.000 (0.000–0.172) 0.653 (0.515–0.791) 0.849 (0.816–0.892) 0.885 (0.831–0.940) 0.200 (0.030–0.371)	
	96		DT 160			STTR9 STTR5 STTR6 STTR10 STTR3	0.000 (0.000–0.073) 0.301 (0.194–0.409) 0.157 (0.059–0.256) 0.429 (0.376–0.607) 0.042 (0.000–0.096)	
Swine	301		Typhimurium	PT AMR	0.7651 0.7378	STTR10 STTR5 STTR6 STTR3 STTR9	0.875 (0.858–0.892) 0.868 (0.848–0.888) 0.875 (0.854–0.895) 0.558 (0.522–0.595) 0.538 (0.485–0.591)	Prendergast et al., 2011
Cattle	544 29	116 178 276 5 6 18	Typhimurium 4,5,12:i:_	–	–	*Xba*l-PFGE 5-loci MLVA PFGE-MLVA *Xba*l-PFGE 5-loci MLVA PFGE-MLVA	0.842 (0.813–0.871) 0.981 (0.977–0.985) 0.991 (0.989–0.993) 0.660 (0.466–0.854) 0.797 (0.681–0.913) 0.797 (0.681–0.913)	Kurosawa et al., 2012
Human	50 5		Typhimurium MDR DT104	PT	0.74	CRISPR1 CRISPR2 *Xba*l-PFGE CRISPR1-2 CRISPR1-2 *Xba*l-PFGE 5-loci MLVA	0.84 0.84 0.87 0.88 0.64 0.38 1	Fabre et al., 2012
Human	1,415		Typhimurium	AMR PP	0.88 0.88	5loci-MLVA	0.98	Wuyts et al., 2013
Human	86	45 37	Typhimurium	–	–	*Xba*l-PFGE CRISPR-MVLST	0.948 0.941	Shariat et al., 2013
Human (63) Animal (119) Human (205) Animal (206)	182 411		Typhimurium 4,[5],12:i:-	–	–	STTR6 STTR5 STTR10 STTR3 STTR9 STTR6 STTR5 STTR10 STTR3 STTR9	0.87 (0.84–0.89) 0.82 (0.79–0.86) 0.60 (0.52–0.69) 0.51 (0.44–0.58) 0.37 (0.28–0.45) 0.78 (0.75–0.80) 0.72 (0.70–0.75) 0.06 (0.03–0.09) 0.13 (0.09–0.18) 0.02 (0.00–0.04)	Barco et al., 2015
Chicken	71		Typhimurium	PP	0.969	*Xba*l-PFGE	0.974	Wang et al., 2015
Human	375		Typhimurium	PT	0.749	*Xba*l-PFGE 5-loci MLVA	0.829 0.867	Lienemann et al., 2015
Human (43) Food (49)	92		Typhimurium	–	–	5-loci MLVA *Xba*l-PFGE ERIC	0.976 0.993 0.983	Almeida et al., 2015
Health swine (22) Environment (5) Human (43)	70		Typhimurium	–	–	5-loci MLVA *Xba*l-PFGE ERIC	0.957 0.996 0.983	Almeida et al., 2016
Human (43) Food (49)	92		Typhimurium	–	–	CRISPR1-2 CRISPR-MVLST	0.906 0.906	Almeida et al., 2017

*AFLP, Amplified fragment length polymorphism; CRISPR-MVLST, Clustered Regularly Interspaced Short Palindromic Repeats-multi-virulence locus sequence typing; ERIC, Enterobacterial repetitive intergenic consensus; MLVA, Multilocus variable-number tandem repeat analysis; MAPLT, multiple amplification of phage locus typing; PFGE, Pulsed-field gel electrophoresis; RAPD, Random amplification of polymorphic DNA; Rep PCR, Repetitive palindromic extragenic–PCR; DI, Simpson's diversity index; 95% CI, Confidence Interval, precision of the diversity index, expressed as 95%; PT, Phage typing; AMR, Antimicrobial Resistance Profile; PP, Plasmid profiling; MDR DT104, Multi drug resistance ST DT04*.

Additionally, Gorman and Adley (2004) and Ghilardi et al. (2006) noticed that PFGE was not sensible enough to discriminate similar phage types (i.e., DT104 and DT104b) once they present the same profile in outbreak surveillance of MDR ST. The authors' explanation for this result is that there is no association with the genetic pattern of chromosomal DNA, once ST DT104 and DT104b differ only in their numbers of lysis reactions to the phage. Gebreyes et al. (2006) also verified that the use of phage typing in some strains showed distinctly different types that were considered clones by genotypic methods. Phage types DT104 and U302, in addition to DT193 and DT12, were clustered within the same genotypic clonal types (Jeoffreys et al., 2001; Liebana et al., 2002b; Lan et al., 2003; Lindstedt et al., 2003; Ross and Heuzenroeder, 2005; Boxrud et al., 2007; Prendergast et al., 2011). Furthermore, Lienemann et al. (2015) reported that when a particular phage type circulating among animals is well known, phage typing approach still is useful for outbreak detection in humans, especially when the analyzed isolates are considered clones by genotypic technics.

In summary, phage typing still signifies a valuable tool especially when ST relatedness strains are involved during the evaluation of an outbreak and food source attribution (Gebreyes et al., 2006; Barco et al., 2012; Lienemann et al., 2015). Moreover, it showed to be a good source tracking for identifying strains in particular countries within specifics phage types (Lienemann et al., 2015). Lastly, to phage typing keep being used by international laboratories and play a role in epidemiological studies and control of ST, a total simplification of its methodology is necessary to enhance its robustness, even though this may result in a discriminatory power decrease (Baggesen et al., 2010).

### Antimicrobial resistance profile (AMR)

AMR is usually cheap and does not demand exclusive equipment and reagents yet the cost are depending on the assay (Barco et al., 2013), but needs to be complemented with another subtype method, whereas alone, the discrimination capacity is unreliable (Miriagou et al., 2006). The higher discrimination power is dependent on the number of antimicrobials tested, the stability, diversity and relative prevalence of the detectable acquired resistance mechanisms (van Belkum et al., 2007). The resistance profiles obtained by the Kirby-Bauer method combined with cluster analysis can provide valuable typing data as a complementary data to other approaches (Miriagou et al., 2006; van Belkum et al., 2007). Some studies showed consistent discriminatory power from AMR in comparison with other techniques. Wuyts et al. (2013) for example, compared the DI of phage typing and AMR and found the same valor (0.88) for both approaches (Table 1). In another comparison, a study between swine asymptomatic and swine associate-disease isolates, the PFGE and AMR methods revealed similar DIs (Perron et al., 2007). In this last group, when only an endonuclease enzyme was tested in PFGE, DIs of both approaches were similar. DI was 0.72 in AMR and ranged from 0.72 to 0.85 in PFGE (Table 1). This close results may be attributed to the massive exclusion of uncertain small bands (probably correlated with plasmids) from PFGE analyses. These results are suggestive of the important role that plasmid-like structures can play in relation to the strain virulence. In contrast, Gebreyes et al. (2006) showed that some AMR pattern analysis had the lowest discriminatory index (0.579) compared to the molecular technics (Table 1). Although this phenotyping method had lower discriminatory power, it has proven useful in understanding the extent of antimicrobial resistance among ST isolates. In this study, phage types DT104 and U302 in addition to DT193 and DT12 with different resistance profiles were also clustered within the same genotypic clonal types.

Many factors contribute to the emergence of antimicrobial resistant microorganisms, but the use of antimicrobials in medicine and agriculture is considered the most important factor (NARMS, 2014a). There is an increasing number of patients with severe infections and cause failure in antibiotic treatment (Giraud et al., 2003; Greig et al., 2015). Hence, the epidemiological surveillance of ST AMR is necessary for prediction of occurrence of resistant populations and effective human treatment (Greig et al., 2015). Giraud et al. (2003) suggested that the ingestion of contaminated foods by this microorganism enables the transmission of it through the food chain. It is known that in pork and poultry industries low levels of bacitracin, chlortetracycline, erythromycin, lincomycin, neomycin, oxytetracycline, penicillin, streptomycin are consistently dispensed in each ton of feed and, over the time and, as consequence, these low doses of antimicrobials confer the ability of microorganisms to develop mechanisms of resistance (EFSA, 2009; Landers et al., 2012; Andoh et al., 2017).

There is a large number of international databases built around antibiograms including data on clinical profiles of isolates and geographical origin that support the use of AMR as a valuable tool in ST study (Miriagou et al., 2006; van Belkum et al., 2007). Links between antimicrobial uses in animal production and food chain with antimicrobial resistance in isolates from human have been mostly documented (Threlfall, 2000; Cloeckaert and Schwarz, 2001; Liebana et al., 2002a; Angulo et al., 2004; Olsen et al., 2004; Best et al., 2007, 2009; Perron et al., 2007; Aslam et al., 2012; DiMarzio et al., 2013; Paranthaman et al., 2013; WHO, 2015; Cui et al., 2016; EFSA and ECDPC, 2016). Nowadays, due the strictly controlling on the nontherapeutic use of antibiotics in food animal production, the AMR has generated considerable attention (Brichta-Harhay et al., 2011). Gebreyes et al. (2009) found *Salmonella* isolates from pigs with a higher prevalence of antimicrobial resistance when compared with human isolates. In the same way, Hoelzer et al. (2010) found a significantly higher frequency of multidrug resistance *Salmonella* isolates (Newport and Typhimurium) among cattle (88%) than human (71%). These results raise the possibility, sustaining the theory that cattle may be one probable reservoir for MDR *Salmonella* and that may represent a source for emerging MDR strains. In contrast, NARMS (2014b) reported that the percentage of MDR ST isolates in human has declined from since 34% in 1996 to 14.5% in 2014. Similarly, the percentage of MDR ST resistance in cattle decreased abruptly from 67% in 2009 to 7.1% in 2014. Declining MDR ST in cattle is the primary beacon behind overall declining MDR ST in human isolates (Medalla et al., 2013).

Although it is essential to know the susceptibility profile, AMR has an important drawback. This approach is not utilized alone for epidemiological correlation between strains (Giraud et al., 2003). Generally, AMR derives from the acquisition of virulence genes carried by plasmids (Ferrari et al., 2011a; García et al., 2014, 2016) and/or transposons transferred between lineages of the same species or between different species, as well as, can also be acquired by efflux pumps (Ferrari R. et al., 2013; Galiana et al., 2014). In the absence of selective pressure, these elements may be lost. Although some resistance mechanisms are very stable, such some mutations situated in chromosomal genes, which confer resistance to quinolones (Ferrari et al., 2011b; Ferrari R. G. et al., 2013), the horizontal gene transfer is easily interchanged between strains (Schulman, 2017). The instability characterized by horizontal gene transfer reduces their relevance for epidemiological purposes. Thus, different strains can develop similar resistance patterns at the same time, as well as, isolates belonging to the same lineage may differ in the sensitivity profile (Angulo et al., 2004; Foley et al., 2006). Another important drawback of AMR method is the need to harmonize methodology and analysis among countries. There is a lack of agreement of the data sets in current surveillance systems, principally between veterinary surveillance and human surveillance. Thus, AMR typing has been less frequently used as a subtyping approach to study the correlation between strains and is not a suitable indicator for outbreak analysis when used alone (EFSA, 2008; Sabat et al., 2013). However, if the surveillances data were truly harmonized (through better standardization antibacterial tested, analytical methodologies used and interpretative criteria) will be possible to be used AMR as a potential data in epidemiological studies and within risk analysis (Silley et al., 2011).

## Genotypic methods

The genotypic methods access the genetic elements from chromosomal and/or extra-chromosomal DNA allowing differentiation among close related strains. They include multiple gel electrophoresis and sequence-based techniques (Sabat et al., 2013; Ngoi et al., 2015). These methods have been used in combination with well-established standard techniques, such as serotyping and phage typing in epidemiological studies, improving the differentiation of strains (Tavechio, 2006). In this review, we summarizes the molecular approaches that have been used in the currently ST surveillance networks and disease outbreak investigations as PFGE, MLVA, MLST, CRISPR, Ribotyping, and WGS (Liu W. et al., 2011; Shariat et al., 2013; Shariat and Dudley, 2014; Campioni et al., 2015; Almeida et al., 2017). Besides, we compared the results obtained with different researches to identify more effective and appropriate method or methods for ST tracing.

### Pulsed-field gel electrophoresis (PFGE)

PFGE was first described in 1984, however, was consolidated to *Salmonella* in the 1990s (Threlfall and Frost, 1990) and still is the most widely used method to identify and track this pathogen in outbreaks (Wattiau et al., 2011; Zou et al., 2013). Although PFGE provides less detailed genetic information such as pathogenicity, virulence or antimicrobial resistance genes (Fang et al., 2010) than WGS, it has been successfully used for more than two decades to type *Salmonella* spp. from human, foods and food-production animals (Wattiau et al., 2011; Zou et al., 2013). This is possible due to the discriminatory power, low cost and high reproducibility of this technic (Table 2; Wattiau et al., 2011). The success of this approach resulted in the development of PulseNet databases of PFGE profiles, which currently stores more than 350,000 PFGE patterns of more than 500 serotypes since 1996 in the United States and Europe (Zou et al., 2013). This method, due to all your attributes, having aid enhance surveillance and epidemiological investigations, is recognized as “Gold Standard” to study *Salmonella* spp. (Swaminathan et al., 2001, 2006). PFGE approach consists of the cleavage of the bacterial genome with restriction enzymes, i.e., *Xba*I, *Spe*I, *Not*I, that recognize few sites along the chromosomal DNA, cutting it off randomly and generating from 10 to 30 restriction fragments ranging from 10 to 800 kb. These large fragments of DNA cannot be separated by electrophoresis conventional, but the orientation of the electric field in PFGE is periodically modified allowing fragments of up to 2 megabases be effectively separated by different sizes (Foley et al., 2006; Fang et al., 2010). As universal molecular weight standard for normalizing, the DNA control from *S. enterica* serotype Branderup (H9812) is used as PFGE fingerprints stander allowing the comparison with a database for several bacterial pathogens (Hunter et al., 2005).

**Table 2:** Most relevant features of the subtyping methods for *Salmonella* Typhimurium.

** 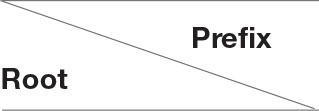 **	**PT**	**AMR**	**PFGE**	**MLVA**	**MLST**	**CRISPR**	**RT**	**WGS**
Bacterial culture required	Yes	Yes	Yes	No	No	No	Yes	No
Typeability	Moderate	Low	High	High	High	High	Moderate	High
Repeatability	Moderate	Moderate	High	High	High	High	Moderate	High
Reproducibility	Low	Moderate^*^	High	Moderate	Moderate	Moderate to high^*^	Moderate	High
DP^a^	Moderate to high	Low to moderate^*^	Moderate^*^	High^*^	Low to moderate^*^	High	Low to moderate	High
Stability	Moderate to high	Moderate	High	Low	High	Moderate	High	High
Level of interpretation	Difficult	Easy	Easy	Easy to moderate	Moderate	Difficult	Difficult	Difficult
Ease of use	Moderate	Easy	Moderate	Moderate	Difficult	Moderate	Moderate	Difficult
High throughput	No	No	No	Yes	Yes	Yes	No	Yes
Cost^b^	Low	Low	Low to moderate	Low to moderate	High	High	Low to moderate	High
Time required^c^	2+	3+	3+	< 2	3+	< 1	2+	< 2^*^
Notes	Needs experience in interpretation	Depends on number of antimicrobials tested; Not utilized alone for epidemiological correlation	Depends on type and number of enzymes; Depends on the strain (Table 1)	Specific to ST	Depends on the number of and the gene choice; Insufficiently DI for use in outbreak investigations	Depends on databases used; Used mainly in France	–	Depends on the sequencer technology and number of strains
References	Threlfall and Frost, 1990; Rabsch, 2007	Giraud et al., 2003; van Belkum et al., 2007	Foxman et al., 2005; Li et al., 2009; Wattiau et al., 2011	Torpdahl et al., 2007; Lindstedt et al., 2013	Foxman et al., 2005; Li et al., 2009; Sabat et al., 2013	Fabre et al., 2012; Shariat et al., 2013	Foxman et al., 2005; Li et al., 2009	Niedringhaus et al., 2011; Weymann et al., 2017

a *DP, discriminatory power*.

b *Per sample for materials, low < EUR 10 < moderate < EUR 100 < high*.

c *Days*.

*PT, Phage typing; AMR, Antimicrobial Resistance Profile; PFGE, Pulsed-field gel electrophoresis; MLVA, Multilocus variable-number tandem repeat analysis; MLST, Multilocus Sequence Typing; CRISPR, Clustered Regularly Interspaced Short Palindromic Repeats; RT, Ribotyping; WGS, Whole genome sequence*.

* *See notes*.

The discriminatory capacity of PFGE depends on the number and distribution of restriction sites throughout DNA, including plasmids, transposons and integrons, which define the number and size of the bands in each profile (Zheng et al., 2011). This index can be enhanced by using different numbers and/or a combination of different restriction endonucleases (Perron et al., 2007; Rivoal et al., 2009; Zheng et al., 2011; EFSA, 2013). As reported by Rivoal et al. (2009), when utilized *Spe*I the DI was 0.71 compared with 0.60 for the *Xba*I enzyme (Table 1). However, due the low DI acquired by both enzymes when used separately, the authors cannot differentiate very close related strains. In contrast, when two or more enzymes are combined, the DI became satisfactory. Additionally, the DI still increases in PFGE associates with other genotyping technique. For example, Malorny et al. (2001) used three enzymes (*Spe*I, *Xba*I, and *Bln*I), and the best discrimination within ST DT104 was obtained by restriction with the *Bln*I enzyme. However, only when plasmid profiling where combined with PFGE patterns from *Xba*I, *Spe*I and *Bln*I the DI was suitable. Higher discrimination ability to subtyping ST isolated from pigs was also obtained by Wang et al. (2015), which isolates were subtyped by the combination of *Xba*I and plasmid profile (Table 1). These are evidence that in general only minor differences occur in the DNA pattern of close related ST strains (i.e., DT104 isolated from pigs) and, PFGE, alone, may not be able to discriminate it (Wang et al., 2015).

Zheng et al. (2011) emphasize in a study the fact that a single-enzyme in PFGE analysis is particularly intangible for ST strains since an 80 or 90% of PFGE characters from any given single-enzyme data set displayed homoplasy. They studied the differences among six enzymes, *Xba*I, *Bln*I, *Spe*I, *Sfi*I, *Pac*I, and *Not*I, and suggest the concatenated use of *Xba*I, *Bln*I, and *Spe*I, which together retained the highest intraenzyme compatibility scores, a fact not observed with *Sfi*I, *Pac*I, and *Not*I. In another study, Son et al. (2013) surveyed 151 ST isolates from multiple host sources with a same concatenated six enzymes utilized by Zheng et al. (2011). The six-enzyme concatenated PFGE analysis was able to sufficiently differentiate the ST strains from different hosts such as chicken, turkey, swine and human. This study also observed that isolates from swine clustered more closely with human isolates of ST serotype, singling out swine as a possible contamination source for this pathogen. It is clear that with combine enzymes, PFGE can associate strain and origin more efficiently. However, in these cases, PFGE may require several days for the conclusion (EFSA, 2013). Despite wide studied, PFGE profiles evaluation remains subjective and requires a better normalization regarding what is the more efficiently enzymes combination.

In summary, although PFGE currently is considerate “the gold standard,” this approach is time-consuming, often taking 3 days to be concluded (Zheng et al., 2011; EFSA, 2013; Paranthaman et al., 2013). Another important drawback refers to its discriminatory capacity once single genetic occurrences, such as integration, single nucleotide polymorphisms, recombination or deletion events can result in differences in the DNA fingerprints (Zheng et al., 2011). Moreover, as described in phage typing section, for some strains, especially the closely related (i.e. ST and *S*. 4,[5],12:i:-; phage types DT104 and DT104b), PFGE is unable in differentiate it (Jeoffreys et al., 2001; Liebana et al., 2002b; Lan et al., 2003; Lindstedt et al., 2003; Gorman and Adley, 2004; Ross and Heuzenroeder, 2005; Boxrud et al., 2007; Brichta-Harhay et al., 2011; Petersen et al., 2011; Prendergast et al., 2011; Fabre et al., 2012; EFSA, 2013; Paranthaman et al., 2013). In this context, PFGE needs to be carefully eligible as genotyping method and due to its intrinsic variability, associations with others subtyping methods may also be required for higher and suitable discriminatory power.

### Multiple-locus variable number tandem repeat analysis (MLVA)

Variable number tandem repeats (VNTR) loci are multiple regions with nucleotides repeats in coding and non-coding DNA sequences present in bacterial genomes (Sabat et al., 2013). VNTRs may vary in size as nucleotide sequence (Ngoi et al., 2015). Even among strains of the same species, the number of copies can be highly variable in VNTR profiles and can range from a few bases to over 100 base pairs in length (Lindstedt et al., 2003; Torpdahl et al., 2007; Ngoi et al., 2015). These sequence patterns enable the development of techniques that utilize variation in the number of tandem repeats to discriminate close related isolates, as occur with multidrug-resistant DT104 strain (Lindstedt et al., 2003; Torpdahl et al., 2007; Fabre et al., 2012).

Multilocus VNTR analysis (MLVA) determines the number of tandem repeats sequences at different loci in a bacterial genome (Lindstedt, 2005). For routine surveillance of *Salmonella*, MLVA presents notable advantages compared to PFGE such as: there are several MLVA protocols for subtyping ST (Lindstedt et al., 2003, 2004; Ross et al., 2009; Chiou et al., 2010; Larsson et al., 2013); the protocol is more simple to execute, it is cheaper and less time-consuming than PFGE (Torpdahl et al., 2005, 2007; Lindstedt et al., 2013); can be completely automated (e.g., pipetting robots; automated machines and analytical software); finally, more easily analyzed and shared among laboratories (Torpdahl et al., 2007; Hopkins et al., 2011; Larsson et al., 2013; Lindstedt et al., 2013; Wuyts et al., 2013). VNTR sizes were measured by agarose gel electrophoresis in its first version, but current analyzes, for size determination, frequently use capillary electrophoresis, since the length of the allele at each locus is well-characterized. MLVA also is replacing traditional phage typing technic in worldwide surveillance networks for *Salmonella* for practical and economic reasons (Lindstedt et al., 2013). Moreover, MLVA generally demonstrates a higher discriminatory power than PFGE to separate closely related strains of ST (Table 1) and has been exhaustively validated due its international repeatability and reproducibility for this serotype (Table 2; Larsson et al., 2013; Pulsenet MLVA, 2013).

The MDR ST DT104, who appeared in the 80s and rapidly became a major problem worldwide, challenged the usefulness of PFGE as the ST “gold-standard” for typing it (Ribot et al., 2002). As described in PFGE section, this strain is epidemiologically close related, exhibits a high degree of homogeneity, as well as, distinct strains display identical PFGE patterns (Boxrud et al., 2007; Brichta-Harhay et al., 2011; Petersen et al., 2011; Prendergast et al., 2011; Fabre et al., 2012). Additionally, several studies had reported that this approach has a discriminatory index greater than PFGE and which can be easily regulated by exclusion or inclusion of a locus to be studied (Best et al., 2007, 2009; Ross et al., 2009, 2011; Chiou et al., 2010; EFSA, 2013; Pulsenet MLVA, 2013; Barco et al., 2015; Liu et al., 2016).

The specificity of this method has been enhanced over the years for ST. In particular, a 5-loci MLVA protocol based on five VNTRs (STTR3, STTR5, STTR6, STTR9, and STTR10) was developed and is widely used in many European laboratories (Lindstedt et al., 2004, 2013; Larsson et al., 2013). In the United States, other MLVA scheme has been used. PulseNet developed a 7-loci MLVA protocol for ST by adding 2 VNTR loci to the 5-loci MLVA scheme (Pulsenet MLVA, 2013). Whatever MLVA scheme used, the arrangement of tandem repeats at an established number of MLVA loci results in a MLVA pattern (Larsson et al., 2013). In this context, Larsson et al. (2013) studied the index of successful comparison of MLVA inter-laboratory involving 20 international laboratories which MLVA typed 15 strains of ST. In 97.3%, the laboratories studied assigned the same MLVA patterns, and this research provided precious information that allows laboratories compare the majority of their MLVA profiles regardless of what hardware and software chosen by the researcher or the primers and the conditions they are using (Larsson et al., 2013).

Several studies had been used this approach to tracing ST to your source due to your high serotype specificity, principally, to phage type DT104. As demonstrated in Table 1, the discriminatory power of MLVA (DI = 1) for phage type DT104 was much more greater when compared to PFGE result (DI = 0.38) (Fabre et al., 2012). García et al. (2013) subtyped *S*. 4,[5],12:i:- isolated from hospitals in Spain and related a high discriminatory power (0.953) of 5-loci MLVA. However, in this study, the DI of PFGE (0.972) was considerate high too. This high DI could be explained by fragments smaller than 30 kb which derive from pUO-STmR/RV1-like plasmids (García, unpublished data) but taken into account to define the PFGE patterns. Additionally, Hoelzer et al. (2010) studied ST and *S*. 4,[5],12:i:- isolated from bovine and humans. They observed to ST that discriminatory power of 4-loci MLVA was significantly higher than PFGE, and both combined showed the highest discrimination. Chiou et al. (2010) evaluated 16 VNTRs with a large number of isolates to assess their allelic diversity, variability and stability to compare the discriminatory power for PFGE and various MLVAs (based on various combinations of VNTRs) and to evaluate the usefulness of MLVA data in delineating phylogenetic structure among ST isolates. They noted that, when the ST isolates were closely related, the discriminatory power of MLVA, independent of the number of loci utilized, was greater than to PFGE, indicating that MLVA was more appropriated to typing strains with this characteristic (Table 1). Even MLVA based on a small set of highly variable VNTRs could exhibit a higher resolving power than PFGE and can be approved to supplement PFGE in routine of surveillance network and outbreak investigation (Chiou et al., 2010). In addition, they concluded that when this approach is based on a larger set of loci it remains an important tool for surveillance and investigation of outbreaks (Chiou et al., 2010). Finally, Almeida et al. (2015, 2016) investigated ST strains using 5-loci MLVA, *Xba*l-PFGE and Enterobacterial repetitive intergenic consensus (ERIC) isolated from human (43) and compare them with 22 healthy swine and 5 swine environment isolates. They conclude that all the genotyping methods were efficient in differentiating the ST strains and some strains isolated from swine and humans may descend from a common subtype (Table 1).

Despite several advantages, the MLVA drawbacks should also be highlighted. Hopkins et al. (2007) studied strains of ST isolated from outbreaks and confirmed that, regardless of VNTR stability, minor changes in loci may occur. VNTRs can mutate quickly; reverse or parallel changes can happen at the same locus generating no recent common ancestry in the same MLVA types (Wuyts et al., 2013; Dimovski et al., 2014). Therefore, before testing, during the culturing and transport, there is always a possibility of changes occur in the strains (Hopkins et al., 2007). Generally, variations only arise in loci STTR5, STTR6, and STTR10 (Hopkins et al., 2007; Petersen et al., 2011) and, one-repeat single locus variants (SLVs) were therefore accepted when evaluating the results of these by the Parameter and Pathogen, European Union (Schjørring et al., 2016; Jensen et al., 2017). In addition, several authors mention, due to its location in a prophage, locus STTR6 as unstable or absent (Litrup et al., 2010; Wuyts et al., 2013). Locus STTR10 is situated in a plasmid and it can also be absent although when present does not vary too much as in STTR6 (Litrup et al., 2010; Dimovski et al., 2014). Especially, for long-term epidemiological investigations, the variability of these loci could prejudices the efficacy of MLVA and, consequently, the dynamics of MLVA loci over a long period (Lindstedt, 2005; Dimovski et al., 2014; Schjørring et al., 2016). In this context, Barco et al. (2015) estimated the diversity among of VNTRs (STTR9–STTR5–STTR6–STTR10pl–STTR3) quantifying the variation of the number of repeats at each locus for *S*. 4,[5],12:i:- and ST. They noted that the DI ranged from 0.37 to 0.87 in STTR9 and STTR6 for ST, respectively and the most variable loci were STTR5 and STTR6. For *S*. 4,[5],12:i:- only STTR6 and STTR5 of 5-loci were polymorphic and the DI were 0.78 and 0.72, respectively (Barco et al., 2015). The discrimination of remaining 3 loci, STTR3, STTR9 and STTR10 had alack, suggestive of insignificant polymorphism (Barco et al., 2015; Table 1). In agreement, the loci with the highest level of diversity were STTR6 and STTR10 followed by STTR5, STTR9, and STTR3 (Prendergast et al., 2011). However, when compared the subtyping approaches, MLVA displayed high diversity raising the possibilities to discriminate between and within diverse phage types and AMR types (Table 1). Several researches also related that STTR10 was the most variable locus followed by STTR5 and STTR6, and the least diversity was found in STTR3 and STTR9 (Dyet et al., 2011; García et al., 2013; Ido et al., 2015). Although theses researches had found variability in three of the five VNTR loci (STTR5, STTR6, and STTR3), MLVA proved to be efficient in distinguishing closely related *S*. 4,[5],12:i:- strains. Additionally, MLVA loci (STTR3, 5, 6, 10) from some isolates were not possible to amplify (Fabre et al., 2012).

Clearly, understanding the evolutionary dynamics of repeat changes and the relationship between VNTR differences and genomic differences are required to infer the real genetic connection between MLVA types for epidemiological typing (Fu et al., 2016). For these reasons, more studies are required to determine the power and limitations of ST MLVA analysis (Dyet et al., 2011). However, once there are more than 30 VNTR loci reported in the literature for ST (Lindstedt et al., 2003, 2004; Chiou et al., 2010; Pulsenet MLVA, 2013), there may be multiples possibilities to improve the assay's discriminatory power.

### Multi-locus sequence typing (MLST)

MLST detects allelic variants in several conserved genes. For *Salmonella*, the seven housekeeping genes *aroC* (chorismate synthase), *dnaN* (DNA polymerase III beta subunit), *hemD* (uroporphyrinogen III cosynthase), *hisD* (histidinol dehydrogenase), *purE* (phosphoribosylaminoimidazole carboxylase), sucA (alpha ketoglutarate dehydrogenase) and *thrA* (aspartokinase+homoserine dehydrogenase) has been developed with fragments of 450–500 bp and actually is one of the most popular genotyping methods (Maiden, 2006; EFSA, 2013). This method examines sequences of multiple housekeeping genes (essential genes which are fundamental for cellular functions) that are involved in primary metabolism of the organism and present in all bacteria within a species. Due to these characteristics, they are not predisposed to large selective pressures, which can lead to rapid sequential changes (Foley et al., 2009; Li et al., 2009). Thus, each strain assigned an allelic profile of seven numbers designated as sequence type (Enright and Spratt, 1999).

Instead that occur in MLVA, MLST is appropriate for long-term studies of bacterial population structures, especially when a high rate of genetic recombination species are subtyped, e.g., ST (Li et al., 2009; Cai et al., 2016). This genetic exchange generated by recombination, which entailed a broad spectrum of bacterial populations, ranging from entirely clonal (recombination does not effectively occur) to non-clonal populations (genetic diversity is randomized by frequent events of genetic exchange) (Pérez-Losada et al., 2013). A great benefit of MLST is that, apart from subtyping bacteria, it provides numerous sequences that can be analyzed in different ways to study the structure of the population and the evolution of bacterial pathogens (EFSA, 2013). Moreover, due to all data produced, this technique is highly reproducible and has an internationally standardized nomenclature, which generates unambiguous results (EFSA, 2013). The allele sequences and sequence type profiles are available in large central databases such as the global MLST database (http://www.mlst.net) which host approximately 7,200 *S. enterica* strains within 1,432 belongs to the Typhimurium serotype (Sabat et al., 2013; Cai et al., 2016).

Several different MLST strategies have been examined in *Salmonella* isolated from the environment, animals or humans (Fakhr et al., 2005; Tankouo-Sandjong et al., 2007; Achtman et al., 2012; Leekitcharoenphon et al., 2012b; Sun et al., 2014; Cai et al., 2016). Moreover, the divergent results among MLST investigations may be due to the distinct protocols adopted as: number of loci that were sequenced and genes studied (Soyer et al., 2009). Some of the MLST protocols are composed exclusively of housekeeping genes (Kotetishvili et al., 2002; Fakhr et al., 2005), while others also include virulence genes (Foley et al., 2006; Sun et al., 2014). A particular MLST scheme, for example, is based on the association of two housekeeping genes with two flagellin genes (*fljB* and *fliC*) (Tankouo-Sandjong et al., 2007). This concatenation aims to establish a uniform pattern of sensitivity applicable at both inter- and intra-serotype levels (Tankouo-Sandjong et al., 2007). As indicated by Foley et al. (2006), MLST and PFGE were able to distinguish a comparable number of patterns, nevertheless, MLST was able to distinguish isolates sharing apparently identical PFGE profiles. In agreement, Kotetishvili et al. (2002) and Sun et al. (2014) determined the relatedness among *Salmonella* spp. isolates using MLST and concluded that it has been efficient. Their studies indicated that MLST was capable of separating in different profiles several strains that in PFGE were clustered together. Additionally, even when only four housekeeping genes were used in the MLST method, this technique was better able to distinguish between Typhimurium serotype isolates than PFGE (Kotetishvili et al., 2002; Sun et al., 2014). The MLST scheme was able to group multiple serotypes of *Salmonella* strains into clusters of genetically closely related, which generally correspond a serotype (Achtman et al., 2012). Concerning the hierarchical level that MLST technique can reach, Leekitcharoenphon et al. (2012b) noted that MLST typing is phylogenetically able to rank up to the species level and even, occasionally, at the subspecies and serotype level but is not discriminatory enough for the source tracking proposal. In order to differentiate strains of the same serotype, PFGE is more appropriate once it analyses genetic variations at the whole genome level (Cooper and Feil, 2004). Thus, for *Salmonella*, it is important to consider multiple parts of the genome content (Achtman et al., 2012). Despite weak in source attribution purposes, some researches suggest that the MLST is an excellent candidate to become a reference in *Salmonella* classification system and may replace the serotyping in this question (Achtman et al., 2012).

Recently, a novel combination was developed for ST in association of two housekeeping genes (*gyrB* and *atpD*) with two flagellin genes (*fliC* and *fljB*) (DiMarzio et al., 2013). Such approach termed MultiVirulence-Locus Sequence Typing (MVLST) in association with another technic, Clustered Regularly Interspaced Short Palindromic Repeats (CRISPRs) has been applied for the subtyping pathogens like ST (DiMarzio et al., 2013; Shariat et al., 2013; Almeida et al., 2017). Altogether, the currently available data suggest that CRISPR-MVLST is capable with a higher discriminatory power than the classical MLST (Sabat et al., 2013). However, the CRISPR-MVLST remains more expensive than whole genome sequence (WGS) but, consequently, the costs will decrease and CRISPR-MVLST analysis will become more habitual (Almeida et al., 2017).

Although highly efficient in structural identification within bacterial populations, MLST has several drawbacks (EFSA, 2013). The major disadvantage of MLST still the high cost (Table 2). The total costs of all reagents required for this technique depends on the number of loci studied, as well as, the country in which this technique is performed (Li et al., 2009). Sabat et al. (2013) estimate that in the European Union, the total costs of an MLST analysis based on seven loci exceed EUR 50 per sample. Moreover, such technique is extremely laborious, time-consuming and may even be insufficiently discriminatory for routine use in outbreak investigations and surveillance of particular pathogens (Sabat et al., 2013).

### Clustered regularly interspaced short palindromic repeats (CRISPRs)

A new family of short sequences repeated (SSRs) in DNA was identified in many prokaryotes, found in approximately 40% of all bacterial species (Jansen et al., 2002), including *Salmonella* (Touchon and Rocha, 2010). There are two main classes of SSRs that can be distinguished: contiguous repeats and interspersed repeats (van Belkum et al., 1997). Generally, these SSRs are part of promoter regions or open reading frames (ORFs), and changes in these SSRs may cause variations in the expression of exposed components on the surface (van Belkum et al., 1998; Mojica et al., 2000). The SSRs were called CRISPR by Mojica et al. (2000) and Jansen et al. (2002), which reflects the characteristic features of this family of clustered regularly interspaced short palindromic repeats. This family is characterized by 24–47 bp DNA conserved direct repeats (DRs) (21 bp in ST) (Jansen et al., 2002), separated by variable 21–72 bp sequences called “spacers” (Mojica et al., 2000). Spacers are short DNA sequences obtained from foreign nucleic acids, such as phages or plasmids that are inserted into bacterial chromosomes to protect them from infection by homologous phages and plasmids (Barrangou et al., 2007). These short DNA sequences can be acquired or lost during evolution of the microorganisms and appear to occur frequently, specifically, in ST (Almeida et al., 2013). Therefore, due to acquisition or loss of these spacer elements, CRISPR may have the ability to distinguish strains comparable to other subtyping techniques, such as PFGE (Fabre et al., 2012; Almeida et al., 2013). The length of a CRISPR array is dependent on the number of these spacers and varies dramatically among different organisms and also among different bacterial serotypes or strains (Fabre et al., 2012). Recently, *Salmonella* isolates have been subtype with this approach (Fricke et al., 2011; Liu et al., 2011a,b; Fabre et al., 2012; DiMarzio et al., 2013; Shariat et al., 2013; Shariat and Dudley, 2014; Deng et al., 2015; Almeida et al., 2017) and studies have reported the presence of two of this loci (CRISPR1 and CRISPR2) in this microorganism (Touchon and Rocha, 2010; Fricke et al., 2011). The discovered of these two loci in the *Salmonella* genome leads the development of a database of the sequence signatures for a number of *Salmonella* serotypes (Weill et al., 2014). Indirectly, it is possible to serotypes identification by comparing the nucleotide sequences of the variable portions of the CRISPR loci deposited in a database (Weill et al., 2014).

CRISPR presents some advantages. The principal is the high-throughput and speed that this method can be completed (<24 h), taking into account the isolation and analysis of DNA (Shariat et al., 2013). The product generated by this technique is extremely robust due to its nature (DNA sequences) but yet depends on interlaboratory or different databases used (Shariat et al., 2013). This approach is also in line with other high-throughput subtyping approaches, including real-time CRISPR analysis and WGS (Fabre et al., 2014). In a 738 isolates investigation with different serotypes, 50 randomly selected clinical isolates were collected to compare three subtype technics: phage typing, PFGE and CRISPR (Fabre et al., 2012). In this study was found for CRISPR1 a DI = 0.84, for CRISPR2 DI = 0.84, and a combination of the two loci, DI = 0.88 (Fabre et al., 2012; Table 1). The same isolates showed a DI = 0.87 for *Xba*I-PFGE profiles. Both methods were more discriminatory than phage typing analysis (DI = 0.74) in the same group of isolates (Fabre et al., 2012). However, the discriminatory power for prevalent MDR DT104 isolates was higher when associated with CRISPR analysis (DI = 0.64) than for PFGE (DI = 0.38) (Fabre et al., 2012). In addition, the 5-loci MLVA method achieved the best discrimination (DI = 1; Table 1) (Fabre et al., 2012). In summary, the study has shown that CRISPR is an adequate molecular method to survey *Salmonella* and has a great potential as an alternative for both serotyping and PFGE.

Recently, it was proposed an approach that has been extensively used to molecular characterization of some *Salmonella* serotypes, including ST (Liu W. et al., 2011). The model combines the two CRISPR loci (CRISPR1 and CRISPR2) with two virulence genes, *fimH* and *sseL*. The *fimH* gene has the function of recognizing the cell-host and *sseL* gene induces inflammation and kills macrophages (Liu W. et al., 2011). These genes alone were effective in discriminating isolates from different serotypes while, with the addition of CRISPR1 and CRISPR2, it was possible to separate isolates within the same serotype. This method became known as CRISPR-MVLST (Liu et al., 2011a). These researches observed that CRISPR-MVLST offered better discrimination than either CRISPR or MVLST alone and showed great epidemiologic concordance among eight out of the nine most common illness causing by *S. enterica* serotypes (Liu et al., 2011a). The studied observed that CRISPR-MVLST accurately grouped isolates according to their specific serotypes, except for ST and its variant, serotype *S*. 4,[5],12:i:-, which were clustered together. However, the authors expected this result, due the virulence genes have only provided accurate identification in different serotypes (Liu et al., 2011a). Liu et al. (2011b) concluded that their method appears to be universally applicable to the most clinically relevant *Salmonella* serotypes, and the protocol CRISPR-MVLST may be a useful subtyping method for tracking source of an outbreak. To test CRISPR reliability, Shariat et al. (2013) investigated the discriminatory power of both CRISPR-MVLST and PFGE among a collection of ST. The discrimination index provided by either was similar; PFGE and CRISPR-MVLST were 0.948 and 0.941, respectively (Table 1). With these results, the authors suggest that CRISPR-MVLST provides sufficient discrimination between outbreak and non-outbreak ST, and can be used in concert with PFGE or alone. The results obtained by this study showed, for the first time, that there was an extremely high level of correlation between CRISPR-MVLST sequence types and PFGE patterns (Shariat et al., 2013). Almeida et al. (2017) characterized by CRISPR-MVLST 92 ST strains isolated from humans and food between 1983 and 2013. Interestingly, comparing PFGE results obtained in Almeida et al. (2015), the discriminatory index (0.993) by this approach was higher than for CRISPR-MVLST (0.906). Besides PFGE has presented greater discriminatory, this proves that both methodologies were efficient in subtyping ST (Almeida et al., 2015).

Despite the success in identifying bacterial subpopulations, a major drawback to CRISPR typing and your variants is that besides expensive, the method has been used mainly in France but not internationally (EFSA, 2013). Nowadays this approach begins to be more widely utilized in reason it possesses great capacity of discriminate populations intra serotypes, so, has been expected in a nearly future that this technique closes an important genotyping lacuna.

### Ribotyping

Ribotyping is also known as restriction fragment length polymorphism analysis (RFLP) based method. When this technique is directed to the encoding ribosomal ribonucleic acid (rRNA) the approach is commonly named as ribotyping (EFSA, 2013). The technique principle is based on the highly conserved bacterial rRNA operons, which is flanked by variable DNA regions (Harvey and Minter, 2005). By the use of restriction enzyme, as *Pvu*II, which is usually used for *Salmonella*, the total bacterial DNA is digested (Bailey et al., 2002). After gel electrophoresis and transference of the fragments to a membrane, they are probed with a region of the rRNA operon to reveal the patterns of rRNA genes (Bailey et al., 2002). The sequence variations in the flanking restriction sites result in a small number of different RFLP banding profiles of the conserved domains of the 16S and 23S rRNA genes that are quite simple to interpret (Christensen et al., 2000). However, due to complexity and technicality, manual ribotyping is not a method of choice to typing pathogens (Tenover et al., 1997). Besides, equivalent results between different laboratories are rare and even minimal differences in the protocol can generate divergent results in the final analysis (Pavlic and Griffiths, 2009).

This approach is capable to differentiates some of the strains classified as same phage types and serotypes (Landeras et al., 1996; Liebana et al., 2001, 2002a,b, 2004; Clark et al., 2003). Nevertheless, researchers have reported that some different phage types strains yet revealed indistinguishable ribotypes (Fontana et al., 2003; Adaska et al., 2006; Lindqvist and Pelkonen, 2007), particularly when compared to PFGE (Eriksson et al., 2005). Comparisons of ribotyping with PFGE are unusual because it depends on what were the enzymes applied for digestion and the population characteristics studied (Jeoffreys et al., 2001; Liebana et al., 2001; Fontana et al., 2003). Additionally, when massive subtyping of *Salmonella* strains is performed, this technique cannot be used for source attribution studies due the low discriminatory power (Table 2; Lagatolla et al., 1996; Jeoffreys et al., 2001), even if the analysis is performed with two restriction enzymes (e.g., *Pst*I-*Sph*I, *Hind*III-*Eco*RV, *Hinc*II-*Pvu*II, *Hinc*II-*Sal*I, *Sal*I-*Pvu*II) (Liebana et al., 2001). Finally, in a specifical region, the technique may be not adequate for local epidemiological or surveillance studies (Lagatolla et al., 1996; Jeoffreys et al., 2001).

Currently, to outgrow these limitations, “riboprinting,” an automated ribotyping, has been successfully applied and requires minimal technical skill in the execution by the operator (Clark et al., 2003). A study evaluated automated ribotyping to determinate their ability to discriminate ST strains isolated from different environmental sources. The results demonstrated the suitability of the automated *Eco*RI and *Pvu*II ribotyping with DI = 0.878 in identification in ST (De Cesare et al., 2001). Moreover, Ranieri et al. (2013) compared riboprinting approach that directly targets O- and H- antigen-encoding genes for their abilities to predict *Salmonella* serotypes. In ST, specifically the variant *S*. 5_ was not identified due the database did not contain the reference sequence, and incorrectly identified the variant 4,5,12:i:-. The major drawback of automated ribotyping is the high costs of reagents and the automated riboprinter. However, this method still has being used principally by commercial food companies in identification and subtyping some food-borne pathogens (Fontana et al., 2003; Pavlic and Griffiths, 2009; EFSA, 2013).

### Whole genome sequencing (WGS)

Whole genome sequencing (WGS) is leading the way regarding expanding our ability to identify and characterize bacteria through the identification of subtle differences between genomes (Wilson, 2012; Salipante et al., 2015). The great advantage of this technique, especially the new generation sequencing, is that the WGS can detect only single nucleotide differences in the genome, which allows the distinction of high clonality strains (Salipante et al., 2015). The cost of high-throughput short-read sequence data has dropped precipitously over the past decade, owing to continued improvements to modern sequencing platforms. The Illumina platform, for example, has experienced a dramatic reduction in cost per Gb of sequence over the past few years - from consumable costs of just over $100/Gb in 2012 to <$20/Gb in 2015 (Niedringhaus et al., 2011; Weymann et al., 2017). In recent years, the decrease of the cost combined with high speed have made this approach an opportunity for it becomes more utilized in large bacterial outbreak investigations, including the use in public health microbiology and diagnostic, such as identification, typing, resistance detection, and virulence gene detection (Didelot et al., 2012; Wilson, 2012; Kwong et al., 2015). *Salmonella* genotyping based on WGS is replacing traditional methods and has proven very effective in identifying the source of outbreaks (Allard et al., 2012; Hoffmann et al., 2016), improved trace-back studies (Octavia et al., 2015a; Hoffmann et al., 2016), predicted antimicrobial resistance (Zankari et al., 2013; McDermott et al., 2016) and elucidating the evolution of some *Salmonella* sub-types (Okoro et al., 2012; Zankari et al., 2013; Dimovski et al., 2014; Leekitcharoenphon et al., 2014, 2016; Deng et al., 2015; Kariuki and Onsare, 2015; Fu et al., 2016; Phillips et al., 2016). In addition, WGS also provides ways to analyze more specific differentiation of strains focusing in genome adaptation. In this context, multiples Blast-based tools have been designed for fast genome screening and comparison to detect mobile elements such as resistance markers, transposons, phage-like elements/remnants and plasmids (Zankari et al., 2012; Cosentino et al., 2013; Carattoli et al., 2014; Joensen et al., 2014). These tools usually are available for free use in websites (e.g., The Center for Genomic Epidemiology) and they rapidly compare by blasting the uploaded genome sequences with the NCBI GenBank database and provide a report with all matches and confidence of each one. The researcher can use these results to quickly identify the mobile elements in the microorganism's genome using any genome analyzer software (e.g., DnaStar, Geneious, CLC Workbench) and proceed with the differentiation of close related strains increasing the discriminatory power for example.

The genome data offered a much higher resolution than the genotyping methods previously discussed here and in particular, a study demonstrated that MLVA lacks the power to reveal the true relationships of the isolates from carries patients (Octavia et al., 2015b). This same research group also demonstrated the utility and resolution of WGS for outbreak investigation of ST by sequencing 57 isolates from five distinct and epidemiologically confirmed source ST DT170 outbreaks in Australia (Octavia et al., 2015a). The results showed the added value of genome sequencing in the investigation of point source community outbreaks of ST gastroenteritis. Their findings revealed that WGS provides the resolution that can clearly define outbreaks, identify the source of an outbreak, suggest unsuspected epidemiological links, and indirectly validate the completeness of epidemiological investigations. Moreover, their data show that public health investigations of ST outbreaks relying on MLVA typing may underestimate the size of the outbreaks (Octavia et al., 2015a). Additionally, Leekitcharoenphon et al. (2014) studied the efficiency of WGS in detection outbreaks utilizing the traditional *Salmonella* typing PFGE as a standard procedure for epidemiological outbreak investigations. As expected, when used to closely related strains (e.g., strains with the same phage type), PFGE was less discriminative than WGS typing (Leekitcharoenphon et al., 2014). The reason is that WGS and analysis using nucleotide difference approaches or single nucleotide polymorphisms (SNPs) are techniques with a higher discriminative capacity for epidemiological studies of ST and might be very successfully applied for outbreak detection (den Bakker et al., 2011; Lienau et al., 2011; Leekitcharoenphon et al., 2014; Hoffmann et al., 2016). A study compared WGS for the surveillance of antimicrobial resistance with the current phenotypic procedures. It was reported an overall phenotypic resistance highly correlated with the presence of known resistance determinants, with genotype agreeing with phenotype in 99.0%, suggesting that WGS might eventually replace or be used with great benefit in combination with phenotypic methods initially for surveillance purposes, but eventually also for rapid clinical diagnosis (Zankari et al., 2013).

Even with complete access to the whole genome of a microorganism and the excellent discriminatory power provided for the SNP analysis, WGS has some drawbacks comparing to other genotyping methods especially in concerns of bioinformatics analysis. Overall, the SNP calling analysis is not effortless and depends on the conversion of a raw output of NGS technology into a final set of SNP and genotype data involves a several numbers of steps each of which contributes to the accuracy of the final SNP and genotype calls (Nielsen et al., 2011). Usually, the informative and easier to use bioinformatics tools for analysis of WGS data are restricted and expensive (Leekitcharoenphon et al., 2012a). Free bioinformatics tools are widely available, but it is not always easy to find the relevant ones or even those that were available a few years ago. Besides, free access software's requires more acquired bioinformatics expertise and normally are more laborious and time consumption (Gilbert, 2004). However, SNP calling is increasingly easy to access, and no more are strict to pre-paid bioinformatics software. The Center for Genomic Epidemiology (http://www.genomicepidemiology.org), for example, offers a free service of SNPs phylogenetic tree from assembled genomes or sets of sequence reads just by a mere upload of data. Among the tools which accept raw sequence reads and performs basics bioinformatics analysis also include snpTree (Leekitcharoenphon et al., 2012a), Nucleotide Difference tree (NDtree) (Leekitcharoenphon et al., 2014) and CSI Phylogeny (Kaas et al., 2014). Interestingly, these techniques exclude a significant proportion of basic analysis and bioinformaticians have suggested conducting phylogenetic analyses based on all loci in a genome, rather than limiting the analysis to SNPs (Ahrenfeldt et al., 2017). The emergence and accessibility of next-generation sequencing has resulted in an exponential increase in the amount of data generated from sequencing (Kwong et al., 2015).

However, there are some barriers to the widespread implementation of WGS by clinical laboratories. This approach has high costs of equipment and there is little expertise in bioinformatics. Furthermore, even if many WGS analyses can be theoretically performed on standard computers, the computational power required to process and analyze great numbers of genomes is necessary (Kwong et al., 2015). Thus, before implementing WGS in routine surveillance, it is still indispensable to compare it with traditional method (i.e., antibiogram, PFGE, MLVA) and define which analytic methodology that might be most suitable for a given study (EFSA, 2013). Nonetheless, such technique will allow, like no other technique, epidemiological studies to be performed, as soon as, an outbreak occurs. It will be possible to determine the relationship between strains and their connection with the results obtained from patients, leading to an improvement in the control of a disease and yours spread (EFSA, 2013).

## Conclusion

Phenotyping methods based on antimicrobial resistance profiling, phage typing and serotyping have been used as a basis of *Salmonella* epidemiology in the past and still have its importance. These techniques are frequently used for epidemiological surveillance. Once the data are obtained from animals, it will be possible to compare them with those obtained from food and humans. Additionally, such techniques combined have sufficient discriminatory power to explore the possible relationship between strains and even infer in the attribution of a source.

Among genotype approaches, PFGE is the most commonly used molecular typing method and still remains as a standard technique for *Salmonella* spp. subtyping in routine surveillance laboratories worldwide due the good data collections, which have been stored in data bases for a long time. However, when this technic is used specifically to subtype ST, a secondary method, e.g., phage typing, should be used as a complement, due to this methodology may not distinguish enough close related isolates. MLVA seems a promising alternative to PFGE subtyping ST, especially to MDR DT104 strains and even others relatedness strains as monovariant *S*. 4,[5],12:i:-, due to its reproducibility, high discriminatory power, possibility of automation and principally its specificity for this serotype. In concern to CRISPR-MVLST, it is a relatively new subtyping approach with few studies performed in ST. However, we discussed here its functionality as an independent or combine subtyping method to subtyping ST and, despite its drawbacks, it could be improved by using WGS as next-generation sequencing. WGS can provide substantial information, including all possible changes in the genome, as well as, provide valuable information on virulence determinants, drug resistance, and genome evolution.

Thus, it is a fact that to compared the results obtained by whatever approach used and know the real relatedness, the epidemiological information of the putative source (veterinary/food isolates) needs to be equated to the final line of the chain: the human isolates. Meanwhile, as saw, a gap still exists between these two objects of study. Some studies include results from different periods of time and/or geographic regions, or simply not take account the occurrence of phenotypes or sources that are important to surveillance and public health. Even with the accuracy of molecular techniques, it may not be possible to determine how closely related the isolates are without an evaluation of the distribution, time periods of studies and diversity of the bacterial population in question. In this review, we presented evidence that there is no ideal method for subtyping ST strains. The advantages and drawbacks of the subtyping methods described here need to be evaluated to select the appropriate approach for more discriminatory power. As discussed here, to elect the ideal subtyping method to track ST source attribution, it is essential to evaluate basically two major issues: variants of interest (i.e., when the outbreak occurred or what year the isolates were collected, the number of isolates, the origin of it) and the resources available.

## Author contributions

RF wrote the manuscript; PP reviewed the draft and improved the scientific level; CC-J coordinated contributions and provided the final draft of the manuscript.

### Conflict of interest statement

The authors declare that the research was conducted in the absence of any commercial or financial relationships that could be construed as a potential conflict of interest.
